# Extra Supplement.—The Nursing Mirror

**Published:** 1888-11-17

**Authors:** 


### The Nursing Mirror.

All Contributions for this Supplement should be addressed "The Nursing Mirror," care of The Editor, 38, Old Jewry, E.C.

### En Passant.

HOMŒOPATHIC NURSES.—The *Bath Herald* has published a special article about the nursing institution connected with the Bath Homœopathic Hospital. It claims that Miss Nightingale has specially praised these nurses, and that the matron, Miss Florence Hampshire, is a most valuable acquisition. We can quite understand that the nurses are valuable, for the money they earn in private work pays the nursing staff of the hospital. All the nurses are ladies, and are engaged for three years, serving as probationers for the first year.

SALOP INFIRMARY.—Miss Oldham seems to be appreciated at her new post. The directors of Salop Infirmary have secured an adjacent building called Stone House, to be opened as a nursing home this month. The final arrangement of the rooms was postponed until the directors could have the benefit of the lady superintendent's advice, which was a delightful piece of courtesy and wisdom. Stone House has been connected with the main building by a covered bridge, the improvements and furniture costing £500.

IN THE NURSE'S ABSENCE.—Another proof of the unceasing watchfulness necessary in nurses is given in the sad death of Miss Amy Cornewall, aged twenty, daughter of Sir George Cornewall, of Marcus Court, Herefordshire. The young lady had suffered from religious mania. She was staying at Malvern, under the care of two nurses; but on Sunday, the 28th ult., she jumped through the window of a room on the second floor, falling a distance of 20 feet, and sustaining injuries which on Friday proved fatal. At the inquest a verdict of suicide while temporarily insane was returned.

CARELESSNESS.—Here are a few facts from France about the awful evils which follow from carelessness in a nurse. On June 26th, 1882, a poor lunatic was boiled to death by a nurse, who put her in a bath and forgot her. At the Hospital Tenon, Mdlle. Devillers, a patient, died through the mistake of a nurse in not giving the proper medicine. A child was burnt alive in this same hospital in June, 1883. At the Hospital Allener a child lately died through the carelessness of the nurse in administering the wrong medicine. In July, 1885, two patients died at St. Louis Hospital from strychnine given in mistake for brandy. This collection of horrors is simply given to serve as a warning. Dwelling ever where the great issues of life and death are daily carried out, a nurse is apt to forget how serious is her position; how fraught with fatal results her slightest forgetfulness or carelessness.

MILDMAY DEACONESSES.—The work undertaken by the large band of Deaconesses who daily sally forth from the Home at Mildmay is so quietly done that few are aware of its magnitude or worth. Here are made up thousands of those small bunches of flowers distributed throughout our hospitals by the Flower Mission; here are sewing classes, mothers' meetings, fathers' meetings, etc. Then there are a hundred nursing sisters, under a lady superintendent, who nurse the Doncaster Infirmary, a hospital at Bethnal Green, a Mildmay Cottage Hospital, hospitals at Jaffa and Malta, an infirmary at Newington Green, convalescent homes at Barnet and Brighton, and such private nursing as they can venture to accept. The hospital at Bethnal Green is an excellent little institution, which ministers to about five hundred patients yearly. There is also a dispensary attached to it, which some thousands of patients visit in the course of twelve months.

ADVANCE AUSTRALIA.—The lady pupils of the St. John's Ambulance Classes at Kew and Williamstown, New South Wales, have formed themselves into an ambulance corps called "The Lady Brassey Ambulance Corps," and are taking steps to open a local hospital. In the meanwhile this corps is at the service of any sick neighbours, and when the hospital is opened the members have volunteered to act as nurses.

NEWS FROM NEWARK.—In the hospital at Newark-on-Trent it has lately become the custom to keep a sufficient staff of trained nurses, not only to supply the needs of the institution, but also to be hired by those in the town and neighbourhood who desire efficient nursing, and who formerly had to send to Lincoln for the purpose. In the parish of Newark-on-Trent exists an ancient charity called St. Leonard's Hospital, which has been placed under a new Scheme by the Commissioners. The new trustees of the charity having considerable discretionary power, have allotted £45 to provide a nurse for the sick poor of the town. This trained nurse is procured through the Town Hospital. Her duty is to look after a certain number of cases, and to work for a limited time in those homes where a regular nurse cannot be afforded.

WIGAN INFIRMARY.—A copy of the regulations laid before the board of the Wigan Infirmary, referring to the proposed Royal Albert Edward Nursing Institute, has been printed. The nurses are to be engaged in private and district nursing, and to be under the control of a lady superintendent. Rule 11., for the private nurses, is good: "Each nurse is distinctly prohibited from in any way interfering in the affairs of the family of the patient, and is in honour pledged not to divulge or to repeat any conversation or otherwise she may hear in or about the family where she is acting as nurse. She is expected and required at all times and under all circumstances to evince self-denial, forbearance, gentleness, and good temper towards the patient." The other rules are much the same as in similar institutions, and are very full and clear. It is evident that a great deal will be expected of Albert Edward nurses, but there is nothing like trying to live up to a high ideal.

KING STORK.—There is an old fable we all knew in the days of our childhood about the frogs who got tired of the inertness of King Log, and prayed for a more vigorous monarch to reign over them. Doubtless there were numerous trials and a few injustices in the kingdom of frogs just then, and so, without much thought, and under evil influence, they deposed poor old King Log, and accepted with croaks of joy the advent of handsome King Stork. The age of inactivity and dulness was passed, felt the frogs, and the age of progress and power had come. But alas for the progress too hurriedly sought—the power placed into unscrupulous hands! King Stork was a beautiful bird; the frogs gazed on his fine feathers with pride, feeling he was all their own. They had placed him in his present position, and now they were to reap the benefits. The conclusion of the fable is sad in the extreme. The king whom the frogs have set up for their own advantage and glory, coolly gobbles up his numerous subjects. How much better off had been the frogs had they remained faithful to King Log, and not been led astray by wild dreams of glory and new laws for their kingdom. Perchance the moral of this fable might well be thought upon by some of our nurses just now; if the flighty ones who are dazzled by the thought of registration by a self-constituted authority, instead of by the managers of the nursing schools where they were trained, could look into the future, perhaps they might behold a King Stork feeding complacently on the bodies of his subjects.

### Original Articles.

### SICK NURSING AMONG THE RICH.

An Amateur's View.

Now, the difficulties which beset this subject are unusually great. To begin with, there is one that meets us in our very title; because, in these topsy-turvy days, who *are* the rich? From my own observation I feel inclined to reply, the skilled artizans in regular work. For certainly, if ample means with very few claims and responsibilities, constitute riches, they are the rich ones amongst us; unless, indeed, we elect to give that palm to the house and estate agents, who, now that selling one's house has become the recognised fashionable pastime of the day, must be amassing considerable fortunes. I do not, however, propose in this article to speak of artizans or of house agents exclusively; or even (here another class of claimants to the title of "rich" flashes into my mind) or even the manufacturers and purveyors of umbrellas, waterproof, and goloshes, who surely this year have reached their *summum bonum* of prosperity. But having said whom I do *not* mean to speak of, I find it not easy to specify whom I do mean. "Educated persons?" No, indeed. With the Education Commission staring me in the face I cannot venture to narrow down this phrase to any one section of the community. Nor do I wish to employ that hackneyed, and never (to my mind) distinctive term of "the classes." So I must even fall back upon an old-fashioned expression, which seems, at any rate to myself, to describe more satisfactorily what I mean, and say—the gentry. Sick nursing, then, among the gentry. I remarked in a former article upon the neighbourly kindness and self-devotion shown in times of sickness by the poor. But far be it from me to assert that theirs is the monopoly of these beautiful qualities. It is true that the gentry are not so often found in each other's houses as voluntary nurses and helpers, but that is the result of the different social economy in which they live. Mother and sisters—busy, hard-working persons though they may be—can generally lay aside their occupations for a while to attend upon the family invalid—(indeed the difficulty most constantly is to prevail upon them to give themselves the least relaxation from such tendance). Servants, sometimes old nurses, are available, and so the actual manual assistance of neighbours is but seldom required. There are, of course, old and tried friends—most of us have at least one such—whom we could tolerate, nay, welcome, in our sick-room; but, as a rule, the matter-of-fact attendance of either family or professional nurse is more soothing to the nerves than the attentions of the most affectionate and best-intentioned of friends and neighbours. The teasing recurrence of wonder whether our temporary attendant has perceived the hole in our bed-room carpet, or whether she is noting the crookedness of the pattern of our mantel-border; the impossibility of explaining politely our "fad" about not having the arrangement of our books altered; anxiety as to whether she has been properly supplied with tea, or wine, etc., etc.; these and such-like trifles would be quite sufficient to destroy all comfort and benefit from her ministrations. We all know this; and so, except in special cases of old acquaintanceship or great need, we do not go into our friends' homes to nurse them, or expect them to do the same to us.

Having a trained nurse from a sisterhood or institution is a very different thing, and, in serious or long illnesses, a very excellent and advisable thing; but as I can only treat of these subjects from "an amateur's point of view," I naturally avoid with all care any discussion of the methods of these professional artistes, lest my own deplorable ignorance should all too evidently appear.

There may be various opinions upon the decided advance which the world has made in the article of cool commonsense *vice* sentiment, retired. But it has, at any rate, been beneficial in divesting the generality of young ladies of the notion that sick nursing consists in putting on a becoming *negligé* costume, and sitting gracefully at the bedside, sprinkling the patient with eau-de-Cologne, or administering grapes. And yet there still prevails in some families—even the "best regulated"—a considerable amount of confusion and a want of method and discipline in time of illness. An ardent affection is entertained for the invalid, and so everybody is anxious to do their utmost in the sick-room. The door is opened about five times in the course of a minute; three or four attendants have heard parts of the doctor's directions, and endeavour to carry them out, irrespective of the rest. The servants are distracted by six separate orders for a basin of broth or a poultice, and the utmost vigilance on the part of the invalid (nursed not wisely but too well) is required to ward off duplicate administrations of each dose of medicine. Then, when night comes, all these warm-hearted individuals are anxious to sit up. Three or four encounter each other in the room, prepared for the vigil, and even when a decision upon this point has been arrived at, the patient and nurse are disturbed by a series of apparitions "just looking in to see how they are getting on," the result of which probably is that when the morning arrives, and the weary night-watcher, unused to the task, is longing to be released; the day nurses, almost equally wearied, are involuntarily detained in the clutches of Morpheus.

Sleepiness is a great stumblingblock to some amateur nurses. We have heard of a gentleman who, on his wife being afflicted with neuralgia one night, consolingly suggested that *he* should go to sleep and try to dream of a remedy likely to do her good. We are not aware that this visionary treatment was found efficacious by the sufferer, but the inventor thereof in the morning denied all knowledge of the transaction.

Happily nowadays the study of the art of nursing has become so much more general, and manuals and lectures on the subject are so much more numerous, that we hope such a state of things as that sketched above is becoming less and less possible. But we should like, on the other hand, to put in a warning word to our methodically-disposed young friends not to allow their orderly cut-and-dried arrangements, their tidy array of bottles and glasses, their hygienic disposal of the window-sashes, and their intelligent timetable—not to allow all this, I say, to absorb the whole of their attention to the exclusion of the patient him- (or her) self. The *un*methodical extreme may be the worse of the two; but let it be borne in mind that stern, undeviating method, a kind of nursing by mathematics—unless specially ordered by medical authority—is somewhat dreary and depressing for the patient. Some invalids, in certain conditions, decidedly object to be "cut-and-dried," and probably their state is so varying that the attention of the nurse ought to be given chiefly to observing their condition, cheering, soothing, or amusing, as circumstances require. A startled awakening from a feverish doze, for instance, would be better calmed by the feeling of a quiet, firm, and cheery presence at the bedside than by an indistinct vision in the distance of a knitted brow bent in intricate calculation over the memorandum-paper which by its clearness and regularity is to electrify the doctor on his next visit. Not that I would say one single word against the indispensable use of method in a sick-room. All that I would convey to young amateurs, whose zeal occasionally outruns their discretion in such matters, is the old wisdom: "These ought they to have done, and not to leave the other undone."

I fear I shall be considered hypercritical, but I should like to register a gentle plea against the system of perpetual cheerfulness which some persons seem to think it necessary to adopt in the character of sick nurse. I know I shall be exclaimed at for this, but pray let it be remembered that I said "perpetual;" and perhaps I shall be better understood if I substitute the phrase "cheering-up-ness" for "cheerfulness." Have none of my readers ever been irritated to the borders of a mild frenzy by the persevering and unalterable determination of some well-intentioned personage to cheer them up, irrespective of "moods and tenses." This sounds like black ingratitude, no doubt, but I am content to bear meekly the imputation if these few words on the subject should chance to save some future invalid from an overdose of that exceesive cheerfulness which, to some natures, is almost more objectionable than extreme gloom. As for real, comfortable, genuine cheerfulness, which can yet be serious (not gloomy) on occasion, no one can bid it a more hearty welcome than I into a sick-room, and in proof thereof I shall conclude with dear Miss Alcott's saying (in her hospital sketches) that it is wise to "look well after the cheerfulnesses of life, and leave the dismals to take care of themselves."

Terra-Cotta.

### Everybody's Opinion.

[*Correspondence on all subjects is invited. No communications can be entertained if the name and address of the correspondent is not given, or unless one side of the paper only be written on.*]

### NURSES AND PRESENTS.

In reference to the paragraph on "Nurses and Presents," in the NURSING MIRROR, November 3rd, will you allow me to say that I am sure the committee of the Bath Nurses Institute must greatly regret having "hurt" the susceptibilities of any "lady" who has benefited by the services of their nurses. They rejoice when grateful patients subscribe to the Benefit Fund, and are sure that the Lady Superintendent would never refuse a patient's request for leave to give her nurse a book, or other small token of thankfulness. "The lady" is, however, misinformed as to the rate of salary paid, and fees received. Two guineas is the fee charged for mental and some few infectious cases, the ordinary charge being £1 5*s.* 0*d.* per week. The *average* wage of the staff nurses is over £26 with complete uniform; none has ever been engaged at £20, which is received by probationers for second year after training. With regard to illness, the "lady" is also misinformed. Nurses who have fallen ill in the service of the Home have always been carefully nursed, and then sent to Convalescent Homes. One was nursed for thirteen weeks at the Home. A Nursing Institute is not, however, a Hospital, but a home where the comfort of the nurse is considered, and she is protected from over work and anxiety.—C. A. LEE, Hon. Sec., Bath Nursing Home.

HANDCLASP writes: You have asked the opinion of the nurses as regards the receiving of presents from grateful patients, and the unfailing courtesy with which you kindly print our small articles induces me to contribute my ideas on this "vexed" question.

It seems a pity that such a subject should need discussing, and not very creditable to us, for there ought to be no two opinions about it. To look at it practically; when a nurse agrees to serve an institution for the sum of say £20, and that only, to nurse rich and poor alike, as she may be called upon, just those very people who can make no return for benefits received, I think it both wrong and disloyal, to say nothing of its being very unbusiness like, to quarrel and grumble in a discontented way at the articles of agreement.

No woman is required by law to be a nurse, nor need she join any institution, or subscribe to any rules and regulations, unless of her own free will; but having done so, the least she can do is to come fully prepared to carry out to the utmost of her ability her part of the agreement in an upright and conscientious manner. Perhaps my readers may imagine I am speaking from a comfortable standpoint of from £50 to £60 a-year, but this is not the case. My salary is £20 a-year, and for the fourth year of service too, and presents are quite as strictly prohibited as at the Bath Institute.

I think the whole system of giving "presents," "gratuities," "tips," "douceurs," "backsheesh," or anything else you like to call them, is rotten to the very core, and is the root of a great deal of evil and discomfort in every land. Only take for example travelling, and its attendant discomforts, unless the porter receives a "tip" on the quiet, for simply doing that which railway officials pay him to do. An amusing incident occurred to me lately. I was returning from a "case" in a first-class carriage, and long before the train stopped I had three porters all striving which could first reach my door. At last one, quite out of breath, panted out, "Where for, miss, where for?" I quietly replied, "Nurses' Institution," and, it is needless to say, they all dropped off like a shot. The expression of their faces afforded me infinite amusement and food for thought too. I may add the guard kept touching his cap at every stoppage on my short journey, and was earnest in his assurances I was not far from B——d. I take a cab, and the man tells me his fare is a shilling a mile. I pay him what he asks, and receive for thanks a surly look, and not infrequently my luggage left outside the gates of the institution. The same thing happens at hotels: you are expressly asked not to "tip" waiters, &c., but do not expect comfort and attention if you carry out this rule, and on the day of departure there is the unmistakable look of expectancy on every face from the head waiter to the "under boots," and the officious manner in which one flies for your rugs, another for your stick, a third flourishes open the cab door, is all very suggestive, most suggestive of pounds, shillings, and pence.

Included in the same category in a more or less degree comes presents to nurses; the patient knows he is paying for the services of his attendant, but if it is not absolutely going into the pocket of that same attendant, he has an uncomfortable sensation that he must do something personally for the nurse, who I am sorry to say not unfrequently reminds her patient that not a penny of what he is paying goes into her pocket, a very plain hint certainly. How much grander and better it would be if the nurse let her patient know and feel, that for the time being at any-rate, his welfare is her interest in life, and that quite independent from necessity, she nurses him for the love, and pity, and beauty of the work itself. Nurses in the past, especially in Roman Catholic countries, were spoken of as "Sisters of Charity," "Sisters of Mercy;" it will be grievous if in the future they are spoken of as "Sisters of a-guinea a-week," grasping money-loving women, whose one idea is to make "a good thing out of it." To me the system seems demoralising and unsatisfactory in the extreme, for I think "nursing," however sentimental it may sound, ought to be undertaken for more than a mere livelihood.

It may not be out of place to relate the following story as it was told to me by the wife of a gentleman I nursed—which makes me blush with shame as I recall it, as it will do all true nurses. I was sent to nurse a case of advanced consumption. The people were comparatively poor, and only had a nurse from sheer necessity. My patient died a few days after, and, on leaving, the poor little wife with loving gratitude—the remembranoe of which I shall never forget—thanked me over and over again for the little I had been able to do for her husband, and was grievously disappointed and astonished at my refusal to accept of a present. She said, "Why, the other nurse we had had been most disagreeable until I gave her a handsome present. True, she apologised to me after I had given it to her, and ended up with these words, 'Well, I would have done more to help you, only I thought it was too poor a house to pay me for my trouble.'"

I have rarely been at a private house where I have not been pressed to receive a present, and occasionally I have given offence at my persistent refusal; but why should patients be offended because the nurse does her duty, "looking for no reward?" and what real respect can they entertain for her, if they find their persuasions coupled with her necessities, will suffice to make her break through a standing rule of her institution. I have known, too, patients offer nurses presents to bribe them to a better performance of their duties, and others who think they can say any kind of rude irritating thing, and believe they entirely "make up" for it if only they give nurse a present, and it is not an uncommon thing for ladies to say, "Oh! well, she did her best, but I made it up to her in other ways." Money should only be of value

"Not for a train attendant,
But for the glorious privilege of being independent."

Far before all presents, a nurse values a little kindly consideration, hours of undisturbed sleep, if possible; kind words, perhaps a few flowers specially gathered for her, an extra hour off duty if the nature of the case will permit, a walk or drive planned for her amusement; or, if patients can afford it, and many of those we nurse can do so, why not keep the nurse a week longer than necessary, till she regains brightness and strength for her next case, or let her have the week at her own home if she would prefer, and the expense not too great. I am sure in the majority of cases this would be far more appreciated than expensive lockets, gold brooches, rings, and costly dresses, all of which are useless and unsuitable in the life of a nurse.

A. E. states: As you invite opinions of nurses on the subject of presents from patients, I should like to give you mine as a nurse, and those I have heard expressed by other nurses at different institutes. Almost without exception they think it "hard on" them not to be allowed to accept more than a very small present, which so frequently takes the form of a perfectly useless book. I say nothing about the rule for not accepting money, because, if patients or their friends will only go the right way about it, they can usually find out something the nurse is in need of or would like: though why, if the nurse's pride is not above it, they should not have money I cannot imagine. One nurse I know well was at an institute where they were only allowed to accept only a very small present; when she knocked up with rheumatic fever, while at a case, she not only had to pay a doctor's bill (the medical man having to drive some miles three or four times a day sometimes to see her), but she had to pay for a nurse. When she recovered the committee or superintendent, whoever had the management of those things, declined to take her back, even to finish the three years for which she was bound. Now, had that nurse had a few good presents of money, it would have helped her through that very expensive time. I only hope that there are few institutions with the same principles. One more reason I would urge for the abolition of this rule is that nurses, unless quite conscientious, will break it, and it will always be broken, more or less, for very often the people of the house do not read the rules until the one in question is pointed out to them by the nurse when she is being offered something she may not take.

HOLIDAY *v.* CONVALESCENT HOMES FOR NURSES.—A NORWICH NURSE is sorry to hear from a Lincoln Nurse that she did not study THE HOSPITAL sufficiently. Had she done so, she would still have had the same opinion that a home for nurses after sickness would be greatly valued by many—not a holiday home, as the Lincoln nurse calls it; far from it. Miss Hicks' plan might be perfect; but how many matrons and lady superintendents study their nurses as she does? I should say she is one of a thousand.—[There we believe our correspondent to be wrong. A large and increasing number of matrons undoubtedly do so.—ED. *T. H.*]

### The Nurses' Bookshelf.

[Books for Review should be sent to The Editor, The Lodge, Porchester Square, W.]

### Antiseptics.*

It is seldom indeed that we have the pleasure of reading such a clear, concise work as this excellent little handbook of Miss Hewer's. There is not a superfluous word anywhere; there is not a single word too little. Every chapter is classified and properly headed, and there is a list of appliances and an index at the end. The subject of antiseptics is one of great intellectual interest, and is too often considered as without a nurse's province. But since it often rests with a nurse to see that the practice is carried out, the theory ought certainly to be made clear to her understanding. Unintelligent work is never satisfactory, and such a simple explanation as Miss Hewer's of a difficult subject, could be mastered by any nurse who chose to take the trouble to read and learn. Miss Hewer, after briefly putting forward her object in the preface, opens with a chapter on the fundamental principles of sepsis and asepsis, pointing out the unrest of putrefaction, and the evil work done by the busy little micro-organisms. Then, turning in the next chapter to consider the cure of this evil, Miss Hewer gives a list of the various antiseptics and disinfectants, and concisely notes the advantages and disadvantages of each. The different dressings are then discussed, and compared with other methods of treatment. Chapter V. is the culminating point of the book. Here we have in detail the duties of the nurse with regard to every case where antiseptics are used. The final chapter on antiseptic midwifery should be read by every monthly nurse. We congratulate Miss Hewer on having produced a book which betrays none of the faults common to the writings of most of her sex.

### Our Nurses.†

This is a book founded on the well-known lectures of Miss Barnett, which she has delivered in many places for the National Health Society. It is addressed more to the public than to nurses, and is written especially for the amateur's perusal. The three divisions of nursing given in the lectures are held up in the book—nursing for life, nursing for recovery, and nursing for death. It is a useful book to give to young ladies who have attended ambulance lectures and wish to remember what they have learnt; it might also prove handy in any house where there was sickness, and the family had undertaken the nursing.

### Presentation.

Lambeth Infirmary nursing staff has presented a handsome spirit stand to Dr. G. L. Rugg, on his resignation of the post of Senior Medical Officer.

### Appointments.

Charing Cross Hospital.—After an intensely-exciting contest, Miss Gordon, now of Liverpool, has been appointed matron of Charing Cross Hospital. Miss Gordon trained at St. Thomas's some years ago, and has since held most excellent appointments. Wherever she has been she has been liked equally by doctors, nurses, and patients, and whether to most congratulate Charing Cross or Miss Gordon is a difficult question. There were many well-known names amongst the candidates for the post, including Sister Rosa and Miss Mollett, and it is certainly no mean victory Miss Gordon has achieved.

Miller Hospital, Greenwich.—The committee of the Miller Hospital are to be congratulated upon their choice of a new matron. The fortunate candidate is Miss Edith Pretty, who is highly spoken of by those who have known most of her work at St. Bartholomew's. She has had considerable experience in the training of probationers, and possesses a knowledge of housekeeping as unfortunately rare as it is valuable. It is a good feature in the training school at "Bart's" that instruction is given to probationers in housekeeping by the superintendent of the Nurses' Home. Miss Pretty, who is the daughter of a medical man, will find the Miller Hospital a very pleasant field of labour, and we wish her all success in her new and responsible work.

St. Mary's Hospital, Paddington, has long been deservedly famous for the excellence of the training which probationers and nurses receive there, and its popularity has increased of late, owing to the able management of Miss Medill, the present matron. We are, therefore, glad to be able to announce the appointment of a St. Mary's Sister, Miss Edith Richards, as matron of the Sheffield Infirmary. Miss Richards had excellent testimonials. She is declared "to be" a good and trustworthy nurse, an excellent organiser, and everything she undertakes she does well." She was eight years at St. Mary's, during five of which she was sister-in-charge of one of the largest surgical wards, and also a superintendent of the operation theatre. She has acted as matron at St. Mary's during Miss Medill's absence, and has also been night superintendent there on several occasions. Miss Rickards is much respected and beloved at St. Mary's, and Sheffield is to be congratulated upon its choice of an officer for a post which is by no means always easy to fill.

Stratford-on-Avon Hospital.—The election of matron for the Stratford-on-Avon Hospital took place on Wednesday last. There were fifty-eight applications. The successful candidate was Miss Grace Sackett, late matron of the Windsor Royal Infirmary. She holds a certificate from Guy's Hospital, where she underwent a course of training. The Stratford-on-Avon Hospital is one of the most picturesque of the smaller institutions, and we hope Miss Sackett will find it a pleasant residence, which will offer her many opportunities for useful work.

### Vacancies.

The following vacancies are announced:—

### Nurses.

Nursing Institution, 96, 97, 98a, Wimpole Street, London, W., Mr. Wilson has several vacancies for thoroughly-trained Nurses.

Croydon Union; £22

Brecon Infirmary; £18.

Cumberland Infirmary.

Oakfield Home for Invalids.

Bristol Hospital for Children and Women; £20.

Home Hospital, Eastbourne.

Birkdale Nurses' Home, Southport.

Samaritan Free Hospital.

Mr. Wilson's Institution, Wimpole St.

Croydon Institute; £22.

St. Pancras Workhouse; £20.

Nottingham and Notts Nursing Institution; several.

Royal Surrey County Hospital, Guildford; £20.

Royal Hospital for Children and Women, Waterloo Bridge Road.

Cheltenham Nursing Institution.

Kent and Canterbury Hospital; £25.

Bridgwater Infirmary; £22.

Guildford County Hospital; £20.

Chester Diocesan Deaconess' Institution; two.

Children's Hospital, Derby.

Nurses' Home, Beckenham.

Nurses' Home, Newcastle-on-Tyne.

Miss Boor's Home, Nottingham.

Swansea Nursing Institute.

Newport Infirmary; £25.

Kingston Home, Hull.

Cardiff Infirmary.

Leeds District Nurses' Home; £25.

Brecon Infirmary; £18.

Canterbury Nurses' Institute; several.

### District Nurses.

Newlands, Ryde.
Newcastle, Staffordshire.

### Probationers.

Croydon Union: £[illegible].

Hospital for Children and Women, Waterloo Bridge Road.

Bath Homœopathic Hospital.

Bootle Borough Hospital; several

Workhouse Infirmary Nursing Association.

Brighton Borough Fever Hospital; £16.

### Ward Assistant.

St. Pancras Workhouse; £15.

### Notes and Queries.

### Queries.

(79) *Lectures on Nursing.*—Can any of your numerous correspondents kindly inform me if it is possible to obtain a good course of written lectures? And where I should write for them?—*Probationer.*

(80) *Childhood's Ailments.*—Where shall I be likely to obtain a good book on the treatment of ailments and diseases of infancy and childhood, and what will the price of it be?—*Mater.*

(81) *Hospital Training.*—Will any one kindly inform me where I can receive hospital or infirmary training on payment of a fee?—*M. Britton.*

(82) *Two Months' Surgical Training Wanted.*—A Private Nurse writes: "I have been trained in a small hospital, and am now a private nurse. Can you inform me through The Hospital if I can be received, and on what terms, in a surgical ward of a large hospital for six week or two months. I have high testimonials."

### Answers

(73) *Hip Disease.*—A. M. should visit the Queen Square Hospital. Hip disease has its origin in several causes—falls, chronic disease, poverty, neglect, etc. Treatment often goes on for years.

(73) *Hip Disease.*—Sir Joseph Lister always keeps his patients in bed twelve months, with good results.—*Sister Q.*

(75) *Addenbrookes.*—There are splendid lectures to the probationers by the medical officers; there are also bandaging classes. The nurses have separate cubicles, and the work is lightened for special probationers. The certificate is given at the end of the year.

(79) *Medicus* is informed (1) the uniform is that of the London Hospital Private Nursing Institution; (2) we make no charge whatever.

* "Antiseptics: A Handbook for Nurses" By Annie M. Hewer. Crosby Lockwood and Sons.

† "Our Nurses, and the Work they Have to Do." By H. C. O'Neil and Edith a Barnett. Ward, Lock, and Co.

### THE DOLL SHOW AT BLOOMSBURY.

THOUGH it had been whispered in hospital circles that the Doll Show at the Children's Hospital, Great Ormond Street, was outgrowing its original proportions; still, few were prepared for the truly gigantic fête which took place last week. The dolls were wonderful enough, but they were only a sort of peg on which to hang concerts, theatricals, and entertainments of every kind. If anyone went to the show and came away unsatisfied, he must have been an extraordinary man. The proceedings opened on Friday at three, with a concert in Mary Ward, in which Madame Belle Cole, Mdlle. Ida Audain, Madame Emily Lawrence, Signor Tito Mattei, Mr. Sydney Beckley, and others, took part. The ward was crowded, a large proportion of the audience being children. The most appreciative applause was awarded to Mrs. Keeley, the well-known actress, who recited some lines specially written for the occasion by Mr. Ashby Sterry.

Delivered with power and pathos, the lines were also appropriate and clever, full of those inimitable little touches which make Ashby Sterry a master of society verse. The funds gathered from the fête are to go towards enlarging the hospital, and, referring to this, Mr. Sterry wrote :—

"I plead for them, I beg you each to bring
A tiny feather for our big New Wing :
Let each one use his thought, his means, his might,
To aid us in our new successful flight !"

And again in the conclusion, where reference is made to days gone by, in which Mrs. Keeley played Jack Sheppard, she said :—

"And I must have your money, for I want their lives."

After the concert the visitors adjourned to the large pavilion, which had been erected on the site of the proposed wing, and here were ten stalls where dolls of every sort and description stared stolidly down on the bright moving crowd. The greatest attraction was the stall for dolls dressed as nurses, and here a lively business was being done. Students seemed specially anxious to secure nurse-dolls and in the uniform of their respective hospitals. There were dolls dressed as army sisters, as Bartholomew probationers, as metropolitan district nurses ; there were whole groups of dolls representing ward-life, or leaning over beds, or leading along little patients. It was strange to see the plump, expressionless countenances of the dolls looking out from under the caps which usually cover such different faces, and really the amount of fringe many of the nurse-dolls indulged in was most unprofessional ! At half-past four the Princess Frederica arrived, and was welcomed by loud strains from the band of boys from the Foundling Hospital. After having visited the wards, the Princess proceeded to the pavilion, where she was presented with a beautiful bouquet by a tiny patient. The prizes were awarded as follows : First prize of £5, Mrs. Basil Montgomery ; 2nd prize of £3, Mrs. Marks ; 3rd prize of £1, Miss Nora Hadwin. Special prizes for dolls dressed in hospital uniform, given by Mr. Barry : 1st prize, a surgical case, Nurse Dennis Legg, of the Alexandra Hip Hospital ; 2nd prize, Nurse Florence Booth, of the Hospital for Sick Children. Prizes for children under fourteen, given by Mr. Arbuthnot : 1st prize, Miss Grosvenor and Miss Margaret Phipps ; 2nd prize, Florence Saunders, aged eleven. During the evening, the band of the Grenadier Guards, under Dan Godfrey's direction, played a selection of music, and a fortune-telling gipsy drove a lively trade. At nine o'clock a most amusing little farce, entitled *The Obstinate Family* was performed by various friends of the hospital, and a doll auction by Mr. Douglas Young wound up the first day of the gigantic show.

On Saturday, the second day of the show, the crowd was quite as great as the first, and the stallholders did a brisk business. In the evening it was scarcely possible to get near the stalls, and if it were practicable to raise £3,000 by a doll show, it would surely have been done at Great Ormond Street last week. Certainly we can now hope to see the builders once more at work on the new wing, and to soon chronicle the opening of more wards for the little sick children. Before the evening closed nearly all the dolls had been sold, and as the list of competitors included over 250 names, the sale alone must have realised a nice sum. Princess Alice of Albany exhibited a very grand doll ; a representation of Queen Elizabeth was also one of the features of the show ; and a Sairey Gamp, most cleverly dressed, excited much amusement. The entertainments on Saturday included two concerts, at the first of which Miss Josephine Webling gave one of her clever recitations, and Miss Fuselle sang Cowen's pretty and appropriate song, "The Children's Home ;" a lecture by Mr. Irving Montague, of the *Illustrated London News*, on the "Wanderings of a War Artist," dealt with the reciter's adventures in Egypt and elsewhere, while pursuing his vocation. The band of the Grenadier Guards was in attendance in the evening, and a grand exhibition of Mrs. Jarley's waxworks amused a large audience. A more diverse and continuous feast of amusement than had been prepared for the patronisers of the Doll Show it is impossible to imagine. The medical and nursing staff of the hospital both did their best to make all pass off with éclat, and the stewards, with their green badges, in which reclined a tiny doll, were busy everywhere ; directing, aiding, and controlling the crowd. Many of the visitors took the opportunity of seeing over the hospital, and as many distinguished names were amongst the list of patrons, it is to be hoped that Miss Philippa Hicks has interested numerous new friends in her little charges. There are few people who can visit a children's hospital and come away unmoved ; there are few people who can forget the picture of a children's ward when they have once beheld it. There is no surer way of securing sympathy and support for a hospital than in welcoming all to come and see the work that is there being done. The short-sighted policy which excludes visitors, and receives with curtness the perhaps sentimental offers of help, is bound to ruin a hospital. Therefore, any entertainment which draws strangers within the walls of a hospital is a wise move. We are sure that the Hospital for Sick Children, Great Ormond Street, will derive great benefit from its Doll Show, not only now, but for many years to come ; and we sincerely hope that the promoters of the show will be rewarded for their efforts, their time, and their trouble, by hearing once more the clink of the mason's trowel as the walls of the new wing rise higher and higher. Dear wooden and wax dollies, with your steadily-smiling faces and unfeeling bodies ; we shall have a greater admiration than ever for you, if you have built a shelter for our wan-faced, stricken, wee children !

